# Study on the Influence of Injection Velocity on the Evolution of Hole Defects in Die-Cast Aluminum Alloy

**DOI:** 10.3390/ma17204990

**Published:** 2024-10-12

**Authors:** Hanxue Cao, Qiang Zhang, Weikai Zhu, Sheng Cui, Qin Yang, Zhibai Wang, Bin Jiang

**Affiliations:** 1 College of Materials Science and Engineering, Chongqing University, Chongqing 400030, China; 202309131291@stu.cqu.edu.cn (Q.Z.); zhuwk18258982009@163.com (W.Z.); 19834502526@163.com (S.C.); 2 National Engineering Research Center for Magnesium Alloys, Chongqing University, Chongqing 400030, China; jiangbinrong@cqu.edu.cn; 3 Chongqing Changan Automobile Co., Ltd., Chongqing 400030, China; yangqin1@changan.com.cn (Q.Y.); wangzb@changan.com.cn (Z.W.)

**Keywords:** aluminum alloy die-casting, visualization, injection rate, hole defects

## Abstract

Aluminum alloy die casting has achieved rapid development in recent years and has been widely used in all walks of life. However, due to its high pressure and high-speed technological characteristics, avoiding hole defects has become a problem of great significance in aluminum alloy die casting production. In this paper, the filling visualization dynamic characterization experiment was innovatively developed, which can directly study and analyze the influence of different injection rates on the formation and evolution of alloy flow patterns and gas-induced defects. As the injection speed increased from 1.0 m/s to 1.5 m/s, the average porosity increased from 7.49% to 9.57%, marking an increase in the number and size of the pores. According to the comparison with Anycasting, simulation results show that a liquid metal injection speed of 1.5 m/s when filling the flow front vs. the previous injection rate of 1.0 m/s caused fractures when filling at the same filling distance. Therefore, the degree of the broken splash at the flow front is more serious. Combined with the analysis of transport mechanics, the fracturing is due to the wall-attached jet effect of the liquid metal in the filling process. It is difficult for the liquid metal to adhere to the type wall in order to fuse with subsequent liquid metal to form cavity defects. With an increase in injection velocity, the microgroup volume formed via liquid breakage decreases; thus the volume of air entrapment increases, finally leading to an increase in cavity defects.

## 1. Introduction

Pressure die casting is characterized by high production efficiency, favorable economic indicators, high dimensional accuracy, and good interchangeability, leading to its widespread application and rapid development in large-scale manufacturing industries [1,2]. Porosity defects are among the most typical defects found in castings produced by pressure die casting, and such defects can significantly reduce the mechanical and processing properties of the die-cast components, potentially resulting in the rejection of the castings [3,4]. The main causes of porosity defects include the shrinkage of the alloy during the solidification process and the presence of gases during production. The primary sources of gas generation during the pressure die casting process include (1) entrapped gases during the filling process; (2) gases produced from mold coatings; and (3) gases dissolved in the molten metal during melting. Among these, the irregular and chaotic flow of liquid metal that entrains gases is the most critical factor contributing to porosity defects. ADC12 is a commonly used aluminum alloy in die casting, and it is widely utilized in practical production. Therefore, it is essential to investigate the behavior of gases during the die casting process of this alloy to reduce or eliminate the formation of internal porosity in the products.

In the die-casting production process, the mold structure and process parameters are the primary factors influencing the flow of molten metal, and they are also the main reasons affecting the formation and distribution of porosity defects. Regarding the impact of process parameters and mold structure on the formation of porosity defects, Hu et al. [5] prepared 6061-SiC composites using a composite casting method and subsequently employed vacuum-assisted high-pressure die casting technology at rapid injection speeds of 1, 2, and 3 m/s. The results indicated that with the increase in rapid injection speed, the thickness of the skin layer significantly decreased. As the wall thickness increased, the porosity of the samples first decreased and then increased. Yi-hu Ma et al. [6] utilized three-dimensional reconstruction technology to characterize the microstructural morphology of Mg-3.0Nd-0.3Zn-0.6Zr alloy castings produced by high-pressure die casting (HPDC). The results demonstrated that with an increase in slow injection speed, the porosity transitioned from an aggregated to a dispersed distribution. Regarding which factor—process parameters or mold structure—plays a more significant role in defect formation, the study conducted by Majernik et al. [7] provides a reference. They investigated these two factors by adjusting process parameters and employing different pouring system structures and concluded that changes in the structure of the pouring system or its structural nodes are related to gas retention within the casting volume and are more beneficial for reducing the formation of porosity defects than adjustments in process parameter settings.

With the advancement of computer technology, numerical simulation utilizing Computational Fluid Dynamics (CFD) and various models has provided a convenient method for understanding the filling process of molds and the generation of defects. Ibrahim et al. [8] analyzed the filling and solidification behavior of molten metal based on numerical simulation methods and performed porosity predictions. The results of actual cast parts validated the accuracy of the numerical simulations, indicating a significant correlation between the numerical simulation of the filling process and the solidification behavior of the castings, thus allowing for accurate predictions of shrinkage cavity locations. Gautam et al. [9] investigated the filling behavior of rheological semi-solid slurries in pressure die casting and found that entrapped air in the overflow zone could lead to the formation of voids when the piston ram speed is relatively high. By comparing the appearance of castings and the simulation results, as well as X-ray inspection results, they concluded that the simulation and experimental findings were in close agreement. Haghniaz et al. [10] compared the flow patterns of metal within the cavity and cavity pressure under different conditions. The flow patterns obtained from numerical simulations of various geometries of flat steel molds were found to be highly consistent with the experimental flow patterns, exhibiting nearly perfect agreement. However, for molds with complex geometries, some minor discrepancies were observed.

The simulation methods used in the aforementioned cases belong to grid-based simulations, while meshless methods, particularly Smoothed Particle Hydrodynamics (SPH), have garnered increasing attention in recent years. Unlike grid-based methods, SPH does not require a mesh to compute spatial derivatives, and its meshless nature eliminates numerical diffusion issues between interfaces, making it suitable for fluid problems involving droplet formation, splashing, and complex free surface movements [11]. Research by several scholars has demonstrated that the SPH method achieves high accuracy in simulating and predicting porosity and shrinkage defects in polymer melt injection molding and casting processes [12,13]. Furthermore, Seydani et al. [14] introduced and designed a universal experimental testing case that can model the gravity casting process in 3D while also allowing for the analysis of fluid dynamic filling processes in 2D and the study of cooling and solidification processes in 1D to validate the results of the SPH method. After comparing the results obtained from the 3D mesh-based commercial software ProCAST 2022 with those derived from the 2D SPH method, it was concluded that the SPH simulation method is more adept at capturing the details of fluid motion.

Regardless of the mathematical model and simulation method employed, experimental validation is necessary to confirm their effectiveness. Water simulation experiments provide a viable approach for such validation [15,16,17,18,19]. According to the principle of mechanical similarity, when the dynamic similarity of water aligns with that of liquid metal, the flow characteristics of the liquid metal can also exhibit a certain degree of similarity. However, the physical properties of water and liquid metal differ significantly, which can greatly impact flow characteristics. For instance, the viscosity of water does not vary significantly with temperature, making it inadequate for accurately reflecting the details of liquid metal filling behavior.

Moreover, there are inherent challenges in comparing water simulation experiments with numerical simulations. Niida et al. [20] utilized water simulation equipment to directly observe mold filling behavior and conducted mold filling simulations using TopCAST (https://www.toyotasystems.com/product/cae/detail/topcast.html, accessed on 10 October 2024). Their analysis indicated that the filling behavior of water and the volume of entrapped air were nearly consistent with experimental results. Although the computational results aligned closely with the experimental trends, some discrepancies were still evident. Consequently, there is a necessity to validate the computational conditions and introduce new air movement algorithms to make the simulations more representative of real phenomena. Liu et al. [21] conducted both water and numerical simulations of flow behavior in a steel ladle, noting that the density difference between the tracer and water in the experiments could lead to significant errors.

In summary, there is an urgent need for a feasible method to directly characterize and study the flow behavior of molten metal within molds. To address this, this paper innovatively adopts a novel dynamic visualization method for die filling characterization, utilizing a specially designed die with a glass window. This setup allows for direct observation and investigation of the filling flow behavior of high-pressure die-cast aluminum alloys within the mold, thereby facilitating the analysis of the mechanisms underlying casting defects and the impact of varying injection rates on the fluidization and evolution of gas-induced alloy defects.

## 2. Experimental Steps

**Figure 1:**
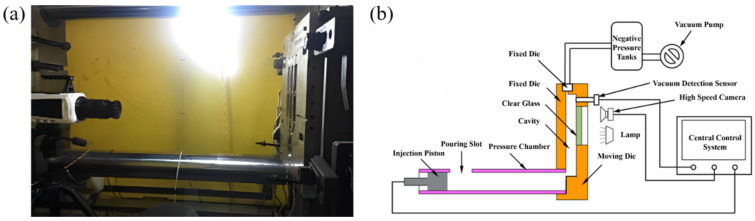
(**a**) DCC630 horizontal cold chamber die casting machine, (**b**) Visual platform diagram.

The die-casting equipment used in this experiment is a horizontal cold chamber die-casting machine, model number DCC630M, as shown in Figure 1. During the experiment, a high-speed camera (the shooting accuracy is 1000 frames per second, with a resolution of 1024 × 1024) was placed on the side of the moving die to film the aluminum alloy solution filling flow process. In order to achieve photography of the experimental process, we have reengineered the die casting machine so that the die casting machine opening, closing, and parts intake port are performed manually. We moved the die casting dynamic machine template to the open mold’s maximum distance position for the convenience of the high-speed cameras, lamps, and lanterns. At the same time, it was connected to the dynamic template clamping electronic scale. The electronic feet were manually adjusted before injection. The die casting machine has clamping signals to ensure that the dynamic template does not close during the injection mode operation. This safety measure is to ensure the safety of the high-speed cameras and shooting experiment. After each shooting experiment, we manually opened the mold, took out the parts, reinstalled the transparent glass on the moving mold, manually closed the mold, and began the next experiment.

**Figure 2:**
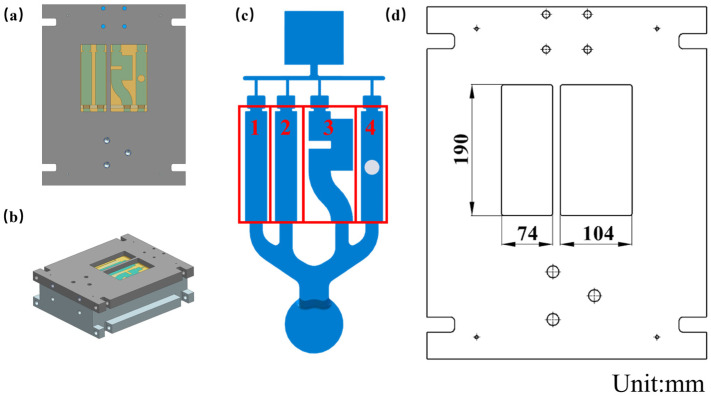
Opening position and size of windows. (**a**) Front view of mold; (**b**) Isometric side view of mold; (**c**) Schematic diagram of test sample; (**d**) Window Size Diagram.

**Figure 3:**
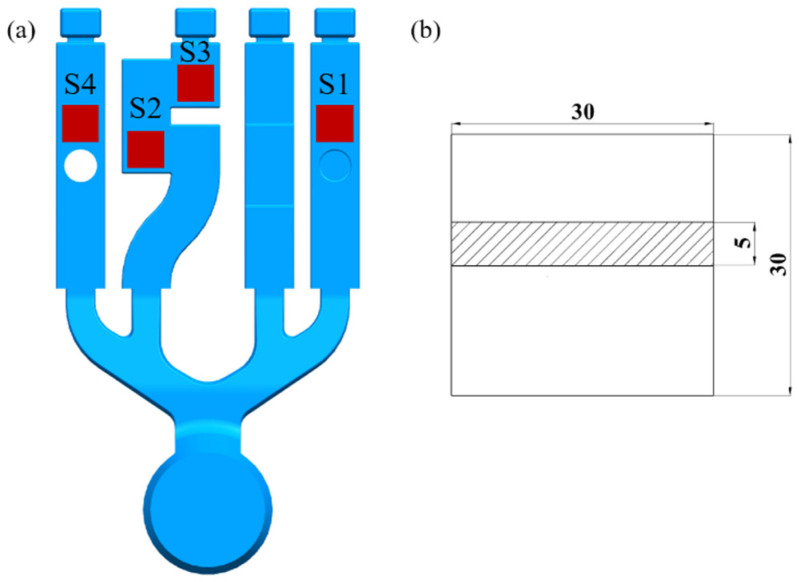
Three-dimensional model of the sample and sampling location (S1, S2, S3, S4). (**a**) Sampling location diagram; (**b**) specimen size.

A mold with clear glass is shown in Figure 2 and Figure 3. The sample die is divided into four common cavity structures, numbered 1, 2, 3, and 4 from left to right. Structure No. 1 is a countersunk hole structure, No. 2 is a stepped boss structure, No. 3 is a bow structure, and No. 4 is a through-the-hole structure. In order to observe the experiment, a window is set on the sample mold, and the window part is assembled with high-temperature resistant glass. Its position and size are shown in Figure 1. The high-temperature-resistant glass material used in all the experiments is high-borosilicate glass.

According to the characteristics of the filling flow observed in the video, samples were taken from the position where the cavity defects were easily generated. These sites are where the porosity is calculated. The three-dimensional model and sampling location of the samples are shown in Figure 3 (sample size is 30 mm × 30 mm × 5 mm):

The samples obtained at different injection velocities were weighed via a static water method to calculate porosity. The model of the density balance used is MH-110E, with a range of 0.0001–120 g, readability of 0.0001 g/cm^3^, weight correction of 100 g, calibration division value of 0.001 g, and actual division value of 0.0001 g. All samples were weighed in air and water, and sample density was calculated according to the following formula:

(1) $$\rho_{p}=m_{1}/(m_{1}-m_{2})\cdot \rho_{w}$$

In the above equation, *ρ_p_* and *ρ_w_* are the density of the sample and the density of water, respectively. *m*_1_ and *m*_2_ are the masses of the sample in air and water, respectively. The porosity of the sample is calculated according to the following formula:

(2) $$P=(1-\rho_{p}/\rho_{wz})\cdot 100\%$$

In the above equation, *ρ_wz_* is the density without holes in the casting. For ADC12, the density is 2.70 g/cm^3^ and the density of *ρ_w_* is 1.0 g/cm^3^.

**Figure 4:**
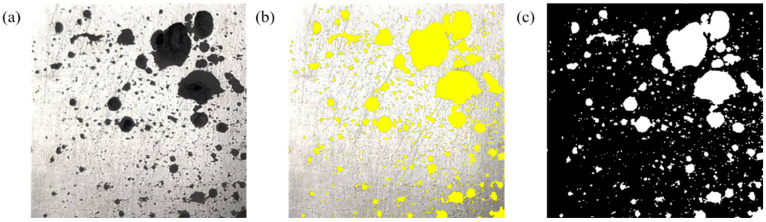
Image-Pro Plus marks the location and size of the hole. (**a**) Original image; (**b**) Coloring image; (**c**) Processed image.

The cross-sectional hole testing software Image-Pro Plus was used to measure the hole defects on the sample’s surface and the effect is shown in Figure 4. The boundary of the defect was captured via a change of light to identify the location of the defect and mark it. At the same time, the required element points were marked, and the redundant element points were ignored according to the needs of the analysis. The qualified holes were then screened out through the measurement function to calculate the number and size of the marked holes.

## 3. Results and Discussion

### 3.1. Dynamic Characterization of the Formation and Evolution of Hole Defects at Different Injection Rates

**Figure 5:**
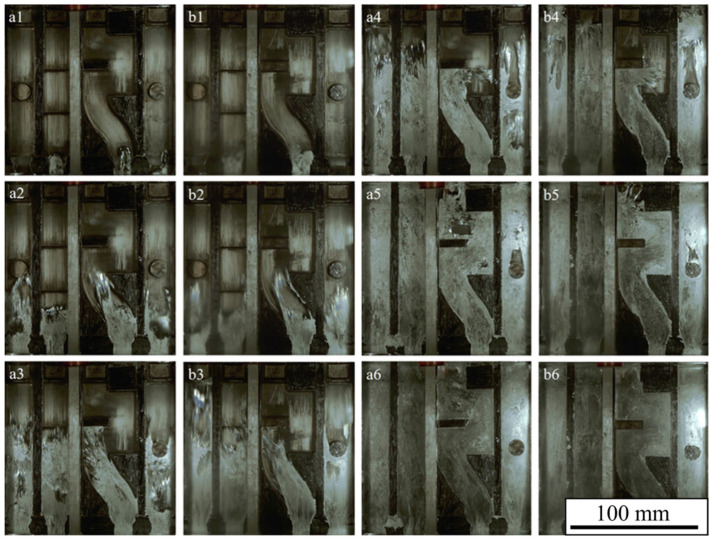
Flow characteristics at different velocities of injection: (**a1**–**a6**) 1.0 m/s, (**b1**–**b6**) 1.5 m/s.

Figure 5 is a comparison diagram of the flow characteristics of samples at a series of filling positions at different injection velocities. a1~a6 are the flow characteristics at different filling positions when the slow injection velocity is 0.2 m/s and the fast injection velocity is 1 m/s (hereafter referred to as the low speed). b1~b6 are the flow characteristics of the filling positions corresponding to a1~a6 when the slow injection rate is 0.2 m/s and the fast injection rate is 1.5 m/s (hereafter referred to as the high speed).

Figure 5 shows that the a1 liquid metal first enters the No. 3 cavity, and the flow state is relatively stable. The liquid metal in b1 was also the first to enter the No. 3 cavity; splashing to a great extent occurred at the flow front when compared to a1.

As the flow advances from positions a2 and b2, splashing occurred as the flow front moved into the No. 1 cavity in a2, therefore producing more cavities. The splashing of the flow front within the No. 3 cavity is intense, the flow front in cavity No. 4 is U-shaped, and the flow front near the sidewall is round. The overall flow state of the liquid metal in b2 is more chaotic than that in a2, and the degree of splashing at the flow front is more severe. The flow front in the cavity of No. 4 is significantly different from that in a2, showing an opposite U-shape.

When the filling reaches positions a3 and b3, the flow front of cavity No. 1 in a3 passes through the boss without any change. In the No. 3 cavity, the flow front collides with the mold wall when it reaches the right corner, resulting in the accumulation of liquid metal. When the liquid metal in the No. 4 cavity flows through the cylindrical boss, it is blocked by the boss to form two metal flows close to the sidewall. The flow front of cavity No. 1 in b3 is close to the filling end. The flow front of cavity No. 3 splashes forcefully, and there is an obvious flow discontinuity phenomenon. Two metal flows behind the boss in cavity No. 4 appear atomized at the flow front.

When the flow reaches the position of a4 and b4, the No. 1 cavity in a4, due to the accumulation of liquid metal in the boss, hinders the subsequent liquid metal flow, which is conducive to filling the void near the inner gate. When the liquid metal in cavity No. 3 flows from the middle of the arch to the sidewall, the flow front diverges. The liquid metal flow front of the two sidewalls in cavity No. 4 has an observable accumulation phenomenon. In cavity No. 1 of b4, much of the liquid metal has entered the overflow groove, and some large voids have been formed at the end of the cavity. The flow front of liquid metal in cavity No. 3 is also divergent, but the degree of divergence is more significant than that of the a4 cavity. The overflow groove in No. 4’s mold cavity is occupied by liquid metal, and many large voids are formed at the end of the filling mold. At this time, the gas trapped in the void is not easy to discharge from the mold’s cavity.

When the filling reaches positions a5 and b5, cavity 1 of a5 is filled. A vortex structure is formed at the upper left and lower right corners of cavity 3, which will entrap part of the gas. There is still a large cavity in No. 4 above the boss structure, and the reflux liquid metal continues to fill the cavity. In b5, cavity No. 1 has been filled, cavity No. 3 only has some small vortex structures at the upper left corner, and cavity No. 4 has a filling morphology similar to a5. a6 and b6 are in a filled state, without residual cavities.

**Figure 6:**
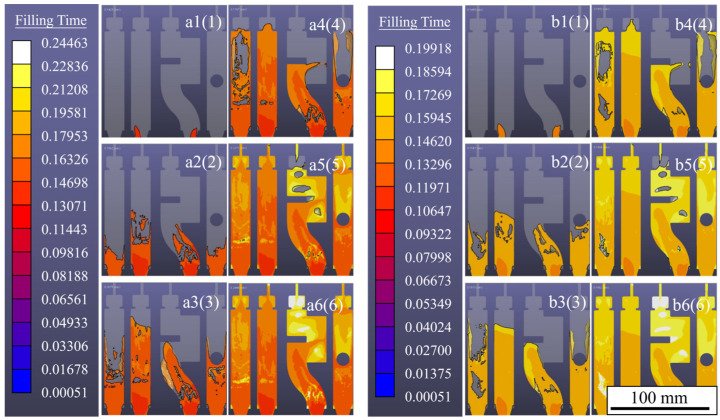
Simulation results of filling flow at different speeds: (**a1**(**1**)–**a6**(**6**)) 1.0 m/s, (**b1**(**1**)–**b6**(**6**)) 1.5 m/s.

Figure 6 shows the simulation results of the flow characteristics at the filling positions corresponding to Figure 5 at different fast injection velocities. a1(1)~a6(6) is the simulation result when the injection velocity is 1.0 m/s, and b1(1)~b6(6) is the simulation result when the injection velocity is 1.5 m/s.

When the liquid metal in a1(1) enters the cavity, the flow front is smooth and round. The flow front in cavity b1(1) is also smooth and round and is different from the broken front in cavity b1.

When the flow reaches positions a2(2) and b2(2), the flow front in a2(2) presents different fragmentation degrees. In cavity b2(2), the flow front of the No. 1 cavity has a broken phenomenon, and the flow front of cavity No. 3 has cavities, which are the same as the actual cavity but slightly smaller.

When the filling reaches positions a3(3) and b3(3), the flow front of cavity No. 1 in a3(3) is attached to the two sidewalls as it moves forward. The morphology is significantly different from that of the actual experimental situation. The flow front of cavity No. 3 is in the shape of an arc, and the liquid metal of cavity No. 4 bypasses the boss to form two metal flows. The dispersion cavity remains under the convex state, which is similar to the actual experimental condition. The flow state of cavity No. 1 in b3 is significantly different from a3 and a3(3). The number of voids in cavity No. 3 is less than that of a3(3).

When the flow reaches positions a4(4) and b4(4), the flow front of cavity No. 1 of a4(4) reaches the overflow tank, which is slightly ahead of the actual position. In cavity No. 3, the liquid metal flows from the middle of the arch to the sidewall in the shape of a font, which is different from the actual dispersion. A considerable accumulation occurs in the flow front of cavity No. 4 near the overflow groove, where the two metal flows tend to converge. The overall flow pattern of b4(4) is very similar to that of a4(4), and the flow front of cavity No. 3 is also in a font shape, which is significantly different from the actual flow pattern of b4. When the flow reaches positions a5(5) and b5(5), the basic shape of cavity No. 1 is completed, and a cavity with a vortex structure is formed at the corner of cavity No. 3 on the upper left and lower right. Among them, a5(5) is close to the actual flow pattern, while the flow pattern of b5(5) is significantly different from the actual flow pattern.

It was found that the simulated results can accurately predict the flow trend, as well as the void and vortex structures generated in the filling process, by comparing the actual filling process with the simulated filling process under the above fast injection conditions. However, the simulation results were earlier than the actual filling, and the actual numerical simulation of the flow front morphology could not be accurately displayed. This may be related to the idealization of the modeling and parameter settings during the numerical simulation, such as the heat transfer coefficient and the material’s physical properties. It is also possible that the meshes are not finely delineated, and the mesh cells are smaller than the volume of particles generated in the actual flow.

### 3.2. Pore Distribution Morphology of the Sample Cross Section

**Figure 7:**
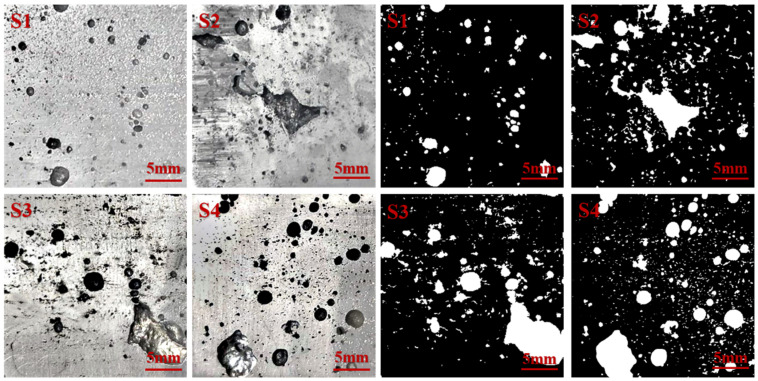
Cross-sectional morphology of specimens with an inner gate thickness of 3.0 mm and an injection velocity of 1.0 m/s.

**Figure 8:**
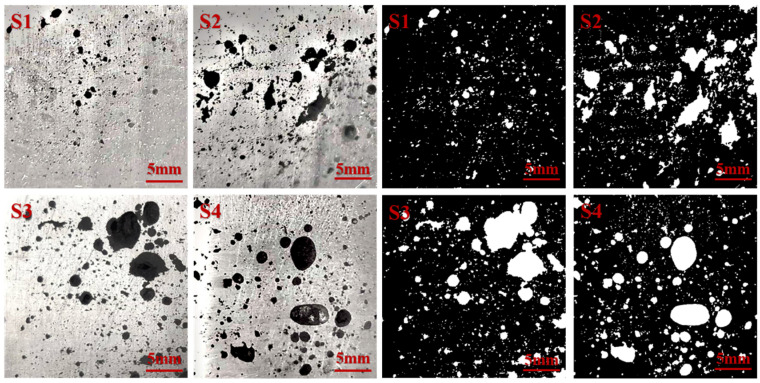
Cross-sectional morphology of specimens with an inner gate thickness of 3.0 mm and an injection velocity of 1.5 m/s.

In high-pressure die-casting, changing the technological parameters will change the flow process of the metal filling. A change in the cavity structure will also change the flow state of the liquid metal, together affecting the defect location, morphology, and size of the casting. Therefore, four positions of different cavity structures were sampled under different die-casting conditions. Figure 7 and Figure 8 and Table 1 and Table 2 show the distribution of voids in the cross-sections of the samples under two different experimental parameters.

Figure 7 and Table 1 show the hole distribution morphology, and the hole defect data at each position S1, S2, S3, and S4 when the inner gate thickness is 3.0 mm and the injection speed is 1.0 m/s. The Figure 7 shows a few scattered S1 positions within the hole. According to Figure 5, the reason is that the metal stamping located at the front of the liquid metal enters the S1 cavity, causing severe breakage. The dispersed metal’s immediate impact on the cavity wall causes a dispersion of the liquid metal onto the cavity, as well as not completely dispelling all the mixed gas. Therefore, a small number of diffuse pores at the S1 position are formed.

In the S2 position, some of the larger, more irregular holes appeared. According to process flow chart analysis, the S2 position, when filled with liquid metal, caused the metal to collide with the right-side wall. After the collision, a reflow was observed, causing an eddy current to form. However, with subsequent liquid metal supplementation, the formation of the eddy currents decreased.

The metal’s flow behavior at the S3 position also led to the emergence of eddy currents, which could not be filled with liquid metal supplementation, causing a large hole to appear after cooling and solidification.

The holes at the S4 position are more dispersed. According to the metal flow process diagram, the front-end is hindered by a convex set in the center of metal, causing an interruption and a liquid metal accumulation in the lower part of the convex platform. The liquid metal in subsequent metal supplementations caused an accumulation in both sides of the upper cavity. In front of the bypass convex sets after the liquid metal is filled to the upper cavity wall, numerous eddy currents appeared during the backflow of position S4, whose radius is significantly larger than that of the S2 position. The subsequent continuous addition of liquid metal makes the radius of the eddy current decrease gradually and finally disappear. The gas that remained in the upper cavity formed scattered holes as the liquid metal cooled.

Figure 8 and Table 2, respectively, show the distribution of the morphology and defect data of the holes at the S1, S2, S3, and S4 positions when the thickness of the inner gate is 3.0 mm and the injection velocity is 1.5 m/s. It was revealed that when compared with those in Figure 7, the S1 location hole size and morphology of the gap are not very large. In the filling flow process, as shown in Figure 5, when the liquid metal filling is fast in cavity 1, front metal atomization occurs, impacting the cavity wall in all directions after reflow. The reflow combination of metal and flow was not intact, and in the middle, a small amount of gas formed after solidification scattering the distribution of the small holes.

Compared with the injection velocity of 1.0 m/s, the hole size at S2 significantly decreases, but a single hole at S2 has a larger area and is uniformly distributed in the center. There are two obvious large holes at S3, which are distributed in the upper right. The S4 position appeared as two ellipsoidal holes. In Figure 8, the liquid metal during the filling process hit the front on the impact of the cavity wall after reflow, toward the center of the convex platform on both sides where the interaction between flow and flow return on the convex platform formed a limited number of small holes. At the end of the filling processes, the gas is not eliminated, and the figure shows that some of the small holes are ellipsoid in shape.

**Figure 9:**
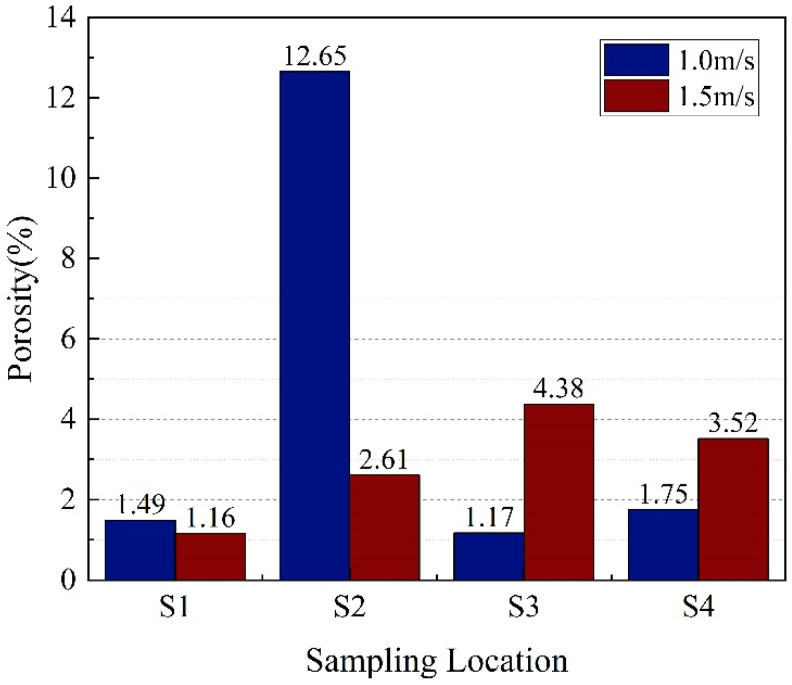
Porosity at different injection velocities at a 3.0 mm inner gate.

The flow state of the liquid metal in the mold cavity has been both the focus and hurdle of the research and will significantly affect the hole’s defect during casting. The injection velocity is one of the controlling factors of the liquid metal flow state. Figure 9 shows the porosity relation diagram of the marked position when other variables are controlled the same and the injection velocity is 1.0 m/s and 1.5 m/s.

Figure 9 shows that when the thickness of the inner gate is 3.0 mm, the sample porosity at position S1 is the same. A seen from Figure 5, the molten metal filling flow process at the two injection velocities is the same, so there is little difference in porosity between the two positions.

**Figure 10:**
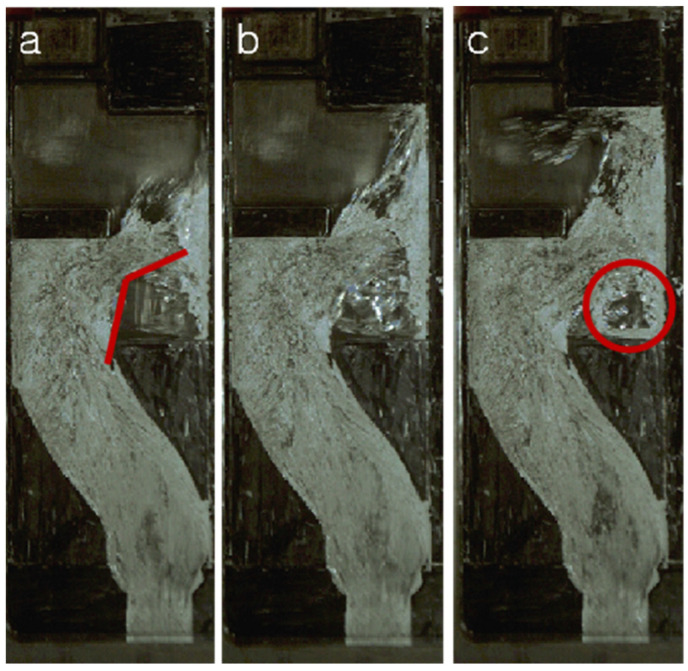
Flow pattern of liquid metal at S2 position at injection velocity of 1.0 m/s. (**a**) t1; (**b**) t2; (**c**) t3.

**Figure 11:**
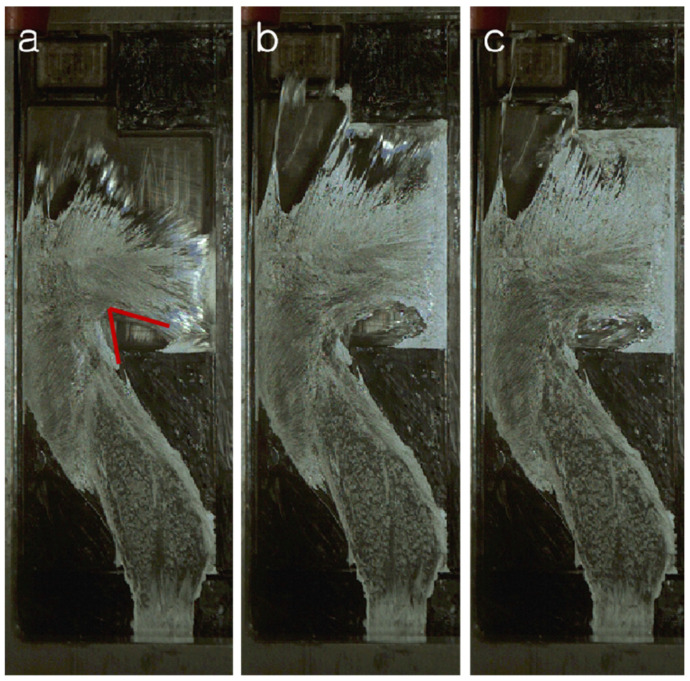
Flow pattern of liquid metal at S2 position at injection velocity of 1.5 m/s. (**a**) t1; (**b**) t2; (**c**) t3.

However, at the S2 position, the porosity of the two is significantly different, with the porosity being more extensive when the velocity is relatively low at 1.0 m/s and reaching 12.59%. To better understand the reasons for the possible difference, Figure 10 and Figure 11 show us the comparison of the flow of the liquid metal at the S2 position.

As shown in Figure 10, when the liquid metal reaches the baffle of the arch cavity, the flow direction of the liquid metal changes. In the red corner of Figure 10a, the change angle is approximately 140°. At this point, the incoming flow, the backflow, and the air in the cavity interact to form a loose vortex flow, as shown in Figure 10b. It can be seen from the figure that some liquid metal with small dispersions has solidified due to a decrease in temperature (the white reflective part in the figure), which adversely affects the liquid metal filling flow’s ability. Due to the reduced flow capacity of the liquid metal and the shear caused by the backflow, incoming flow, and air, the liquid metal will generate vortices, forming a huge hollow cavity at position S2. Its position and size are marked by a red circle, as shown in Figure 10c. Therefore, when the injection velocity is 1.0 m/s, the porosity of the sample is more significant. However, when the injection velocity is 1.5 m/s, the flow state will be significantly different from that of 1.0 m/s injection velocity. As shown in Figure 11, when the liquid metal impacts on the bow baffle at a high speed, the transformation direction of the liquid metal is shown in Figure 11a, with an angle change of more than 90°. The angle formed via the liquid metal at the front and the back end is only about 45°. Compared with the speed of 1.0 m/s, the liquid metal flow’s ability is much stronger. The liquid metal at the front end hits the wall of the right cavity quickly and then turns around and rushes back, forming an elliptical cavity with the liquid metal at the back, as shown in Figure 11b. Due to the fast filling speed of liquid metal, it is subjected to a significant impact force. A large amount of the liquid metal solidification phenomenon is not witnessed in the video. At this time of this experiment, the liquid metal still has good flow ability at the S2 position, so that the subsequent liquid metal will fill the space in the ellipse. Finally, the central part will be filled, forming a small number of hole defects.

The morphology of S3 is similar to that of S2. However, since S3 is at the back end of filling S2, the influence of the injection velocity on the liquid metal flow at S3 will be significantly weakened when the liquid metal flows into S3. It was observed through the video that at the S3 position, the flow morphology differences between the injection velocity of 1.0 m/s and 1.5 m/s are not apparent, so the porosity difference at the position of S3 is not significant. The porosity at the S4 position is larger when the injection velocity is 1.5 m/s.

## 4. Kinetic Analysis of Hole Defect Formation

**Figure 12:**
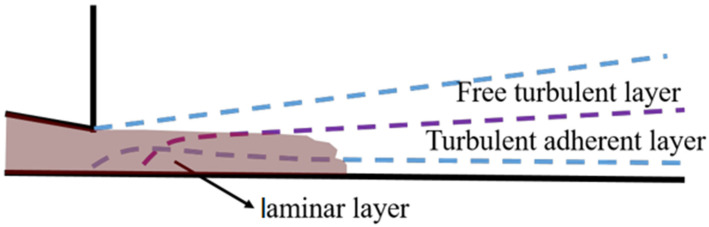
Wall jet model.

According to the transport principle, the flow of liquid metal from the inner gate into the cavity can be simplified by the model shown in Figure 12. The liquid metal thickness at the initial filling stage is consistent with the thickness of the inner gate. The metal flow can be divided into three areas in the direction of thickness: The bottom laminar flow area, whose flow is affected by the viscous force due to the influence of the boundary layer; in the middle is the adherent turbulence area, where the velocity of each flow area is the highest. The upper layer is the free turbulent layer because it has not been affected by the molded wall. The bottom layer and middle layer can be regarded as the turbulent boundary layer, and the upper layer is treated as the turbulent jet. Jet entrapment is due to the discontinuity of the velocity’s stability producing certain fluctuations and developing vortices that cause turbulence so that the static fluid around it will be sucked in. Because the bottom layer and the middle layer are close to the wall, the flow is restricted by the wall’s surface, and the surrounding medium cannot be sucked in, resulting in a large velocity gradient. The increase in kinetic energy and the effect of friction force decrease the static pressure; thus, the pressure difference between the upper and lower sides of the jet is formed, making the jet bend and stick to the wall surface, forming the wall-attached effect. The adhesion wall of liquid metal is difficult to fuse with the liquid metal, so it easily forms cavity defects.

Combined with the dynamic analysis, the liquid jet breakage is caused by disturbance, which develops in the form of surface waves until the liquid cannot bear the deformation force and leads to breakage [22]. At a low jet velocity, the interaction between the liquid and the surrounding gas promotes the growth of long- and small-amplitude disturbances on the surface of the liquid, leading to liquid breakage. For high-speed liquid jets, the fracture is considered to be the result of short wave unstable growth; with an increase in velocity, the micro community volume formed by liquid breakage will gradually decrease.

From the perspective of dynamics, the attraction between molecules in the fluid is balanced in all directions. However, for the fluid at the interface, due to its discontinuity, the cohesive force of the fluid molecules on or near the surface is unbalanced. For a micro-agglomerate in the fluid, the surface tension can keep it continuous. When the resultant force of the external force on the micro-agglomerate is opposite to the surface tension and greater than the surface tension, the micro-agglomerate will protrude and elongate from the free surface. With an increase in the resultant force, the micro-agglomerate will detach from the free surface. When the volume of micro-agglomeration is large, macroscopically, it is broken or splashed, and when the volume of micro-agglomeration is small, it is macroscopically is atomized. The process of the microclusters falling off from the free surface is a process of increasing surface area and energy. Only when the internal energy condition is satisfied can the microclusters fall off from the free surface and show different forms with the energy. The fluidic characteristics of liquid can be expressed as the dimensionless Reynolds number Re, Weber number *We*, and Onneszog number *Oh*, which can be expressed as:

(3) Re=ρudµ    We=ρu2dσ    Oh=µρσd=WeRe

**Figure 13:**
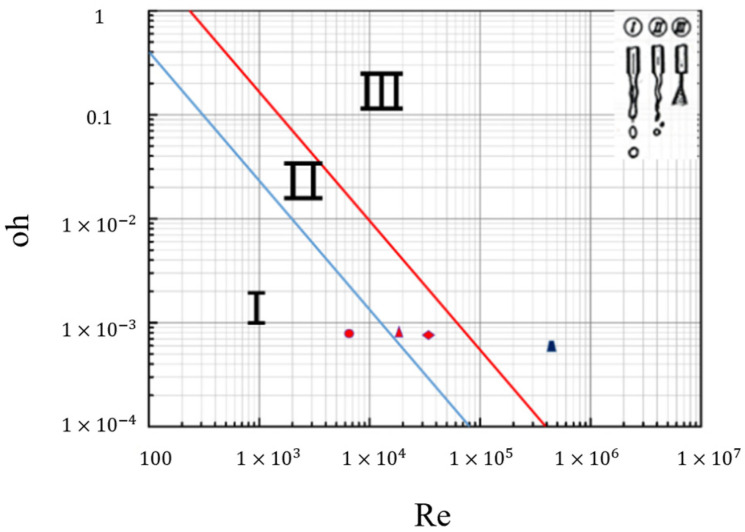
Diagram between Oh and Re.

Types: *σ*, *ρ*, *μ*, *u*, and *d* represent the surface tension, density, viscosity, flow velocity, and characteristic diameter of the fluid, respectively. Re represents the specific gravity of the inertial force and viscous force, *We* represents the specific gravity of the inertial force and surface tension, and *Oh* represents the influence of viscous force on inertial force and surface tension. The relationship between the stability of liquid flow and Re and *Oh* was analyzed. The liquid flow was divided into three different types according to the value of Re and *Oh*: splashing, wavy collapse, and atomization (as shown in Figure 13).

In this test, taking into account the temperature decrease in liquid metal in the chamber and the actual measurement, the temperature of the liquid metal in the cavity is 630 °C, the corresponding density ρ of the ADC12 liquid alloy at this temperature is 2500 kg/m^3^, the viscosity coefficient μ is 0.00167 Pa·S, the surface tension σ is 0.708 N/m, and the characteristic diameter D is the thickness of the inner gate, 3 mm. The velocity of the liquid metal entering the inner gate is 3.2 m/s at a rate of 1.5 m/s injection and 1.25 m/s at the rate of 1.0 m/s injection. The corresponding We values are 108.5 and 16.6, respectively. When the number of We increases, the jet breaking length becomes smaller, while the velocity is proportional to We. Therefore, the greater the velocity of the liquid metal entering the cavity, the sooner the liquid metal ruptures. The velocity of the liquid metal flowing through the cylindrical salient is approximately 18 m/s, and the characteristic diameter d is 5 mm. According to Equation (3), the corresponding Re = 22,500 and Oh = 0.035 can be calculated, which corresponds to the atomization zone (III) in Figure 13. The liquid metal fills the cavity in the form of fog, which can interact with the air to form bubbles, and the gas cannot overflow, thus forming the hole defects.

## 5. Conclusions

In this paper, a visual dynamic characterization experiment of die-casting filling was innovated. The influence of different injection rates on the formation and evolution of defects in the alloy flow patterns, gas-induced properties, and the mechanism of defect generation was analyzed. The porosity of the sample was measured and calculated. Combined with the dynamic characterization of the evolution process of aluminum alloy hole defects under different injection velocities, the following conclusions were drawn:

1. Visualized dynamic characterization of die-cast filling can be observed during the process of liquid-metal liquid cavity filling. Through this experiment, some flow behaviors can be observed directly, such as the process of fluid crushing and air trapping. When the filling speed changes, the numerical simulation of the filling flow trend and the position of the cavity are consistent with the actual filling process. However, the splashing pattern at the flow front is quite different from the observed effects.
2. The filling flow law of liquid metal in the cavity of the arch structure confirms the theory of spray filling by Frommel. When liquid metal enters the cavity from the inner gate, it moves forward in a straight line, and backflow occurs when the flow front collides with the wall of the cavity. Due to the continuous angle structure, a vortex structure is formed at the upper left and lower right corners of the specimen by the return and the incoming flow.
3. Combined with the dynamic analysis, it was found that the liquid metal with high-speed filling is difficult to fuse with the liquid metal from the front end due to the wall jet effect, which leads to the formation of the hole defects. When the injection velocity of liquid metal was 1.5 m/s, the flow front fracture occurred earlier than that of the 1.0 m/s injection velocity. Moreover, under the same filling distance, the breaking and splashing degree of the flow front was more significant, leading to increased air entrapment. The final average porosity increased from 7.49% to 9.57%, and the number and size of the pores also increased significantly.
4. Based on the above analysis, the following measures are proposed to improve porosity through mold structure optimization: 1. Avoid the presence of large flat surfaces in the casting by altering the flow direction of the molten metal to mitigate the wall jet effect. 2. If large flat surfaces are necessary, small protrusions or depressions can be added to the surface to disrupt the stable flow of the underlying molten metal, thereby promoting the integration of the molten metal adhering to the mold walls with the freely flowing molten metal. 3. Introduce flow guiding plates or tubes within the mold to change the direction of fluid flow, allowing for a more uniform flow pattern near the wall. 4. Consider the fluid flow characteristics during mold design, opting for streamlined or curved shapes to minimize fluid stagnation and uneven flow near the wall.
5. This study investigates the effects of filling speed and mold structure on the flow of aluminum alloy filling and the formation of pores. Additionally, it compares the differences between actual filling flow and numerical simulation results. Future work will focus on exploring the impact of temperature distribution on flow and pore formation. Furthermore, to ensure that the numerical simulation results are as close to reality as possible, experiments will be conducted to optimize the settings of numerical simulation boundary conditions and propose a reasonable physical model, providing more reliable analytical support for engineering practice.

## Figures and Tables

**Table 1:** Distribution of surface defects in samples with an inner gate thickness of 3 mm and an injection velocity of 1 m/s.

Location of the Experiment	The Total Area of the Hole (mm^2^)	The Percentage of Holes (%)
S1	8.7857	0.98
S2	76.4286	8.49
S3	71.7021	7.97
S4	112.7857	12.53

**Table 2:** Distribution of surface defects in samples with an inner gate thickness of 3 mm and an injection velocity of 1.5 m/s.

Location of the Experiment	The Total Area of the Hole (mm^2^)	The Percentage of Holes (%)
S1	23.8785	2.65
S2	65.7142	7.30
S3	95.7242	10.64
S4	159.2857	17.70

## Data Availability

The original contributions presented in the study are included in the article, further inquiries can be directed to the corresponding author.

## References

[1] Wang G.G., Weiler J.P. (2023). Recent developments in high-pressure die-cast magnesium alloys for automotive and future applications. J. Magnes. Alloys.

[2] Yang Y.-T., Qiu Z.-Y., Zheng Z., Pu L.-X., Chen D.-D., Zheng J., Zhang R.-J., Zhang B., Huang S.-Y. (2024). Al-enabled properties distribution prediction for high-pressure die casting Al-Si alloy. Adv. Manuf..

[3] Cleary P., Ha J., Alguine V., Nguyen T. (2002). Flow modelling in casting processes. Appl. Math. Model..

[4] Kang H.J., Yoon P.H., Lee G.H., Park J.Y., Jung B.J., Lee J.Y., Lee C.U., Kim E.S., Choi Y.S. (2021). Evaluation of the gas porosity and mechanical properties of vacuum assisted pore-free die-cast Al-Si-Cu alloy. Vacuum.

[5] Hu Q., Guo W., Xiao P., Zhao H. (2021). Effects of Fast Shot Speed and Wall Thickness on the Microstructures and Mechanical Properties of the High Pressure Die-casting 6061-SiC Composites. Metall. Mater. Trans. B-Process Metall. Mater. Process. Sci..

[6] Ma Y.-H., Yu W.-B., Zhou Y.-Q., Xiong S.-M. (2021). Influence of different high pressure die casting processes on 3D porosity distribution of Mg-3.0Nd-0.3Zn-0.6Zr alloy. China Foundry.

[7] Majerník J., Gašpár S., Husár J., Paško J., Kolínský J. (2021). Research and Evaluation of the Influence of the Construction of the Gate and the Influence of the Piston Velocity on the Distribution of Gases into the Volume of the Casting. Materals.

[8] Ibrahim M.D., Mohamad M.R., Roslan L., Sunami Y., Lam S.S. (2018). High Pressure Die Casting Porosity Defect Analysis and Experimental Validation for Power Steering Columns and DVVTs. Lecture Notes in Mechanical Engineering.

[9] Gautam S.K., Roy H., Lohar A.K., Samanta S.K. (2023). Studies on Mold Filling Behavior of Al-10.5Si-1.7Cu Al Alloy during Rheo Pressure Die Casting System. Int. J. Met..

[10] Haghniaz F., Delbergue D., Côté R., Demers V. (2023). Mold filling behaviour of LPIM feedstocks using numerical simulations and real-scale injections. Powder Metall..

[11] Hu M.Y., Cai J.J., Li N., Yu H.L., Zhang Y., Sun B., Sun W.L. (2018). Flow Modeling in High-Pressure Die-Casting Processes Using SPH Model. Int. J. Met..

[12] Ren M., Gu J., Li Z., Ruan S., Shen C. (2022). Simulation of Polymer Melt Injection Molding Filling Flow Based on an Improved SPH Method with Modified Low-Dissipation Riemann Solver. Macromol. Theory Simul..

[13] Niu X.F., Zhao J.Y., Wang B.J. (2019). Application of smooth particle hydrodynamics (SPH) method in gravity casting shrinkage cavity prediction. Comput. Part. Mech..

[14] Zarbini Seydani M., Krimi A., Bedel M., Khelladi S., El Mansori M. (2023). A 2D filling and solidification benchmark test: Validating smoothed particle hydrodynamics (SPH) simulations for sand gravity casting. Int. J. Adv. Manuf. Technol..

[15] Saeedipour M., Schneiderbauer S., Pirker S., Bozorgi S. (2014). A Numerical and Experimental Study of Flow Behavior in High Pressure Die Casting. Magnesium Technology, Proceedings of the 2014—TMS 2014 143rd Annual Meeting and Exhibition, San Diego, CA, USA, 16–20 February 2014.

[16] Kulasegaram S., Bonet J., Lewis R.W., Profit M. (2003). High pressure die casting simulation using a Lagrangian particle method. Commun. Numer. Methods Eng..

[17] Homayonifar P., Babaei R., Attar E., Shahinfar S., Davami P. (2008). Numerical modeling of splashing and air entrapment in high-pressure die casting. Int. J. Adv. Manuf. Technol..

[18] Bi C., Xiong S., Marquis F. (2016). Development of a Full PLIC-VOF Method for Mold Filling Simulation of High Pressure Die Casting Process. Proceedings of the 8th Pacific Rim International Congress on Advanced Materials and Processing.

[19] Sakuragi T. (2013). Mould filling simulation with consideration of surface tension and its application to a practical casting problem. Cast Metals.

[20] Niida A., Maeda Y. (2020). Observation of Air Entrapment during Mold Filling of Die Casting Using Water Model Experiment for Mold Filling Simulation. Mater. Trans..

[21] Liu J., Zhou P., Zuo X., Wu D., Wu D. (2022). Optimization of the Liquid Steel Flow Behavior in the Tundish through Water Model Experiment, Numerical Simulation and Industrial Trial. Metals.

[22] Lin S.P., Reitz R.D. (1998). Drop and spray formation from a liquid jet. Annu. Rev. Fluid Mech..

